# Supplementary material for: Antifungal Properties of Two Volatile Organic Compounds on Barley Pathogens and Introduction to Their Mechanism of Action
Source: Int J Environ Res Public Health. 2019 Aug 10;16(16):2866. doi: 10.3390/ijerph16162866 (PMC6720319; doi:10.3390/ijerph16162866)
Supplement: Supplementary file 1 [file ijerph-16-02866-s001.pdf]

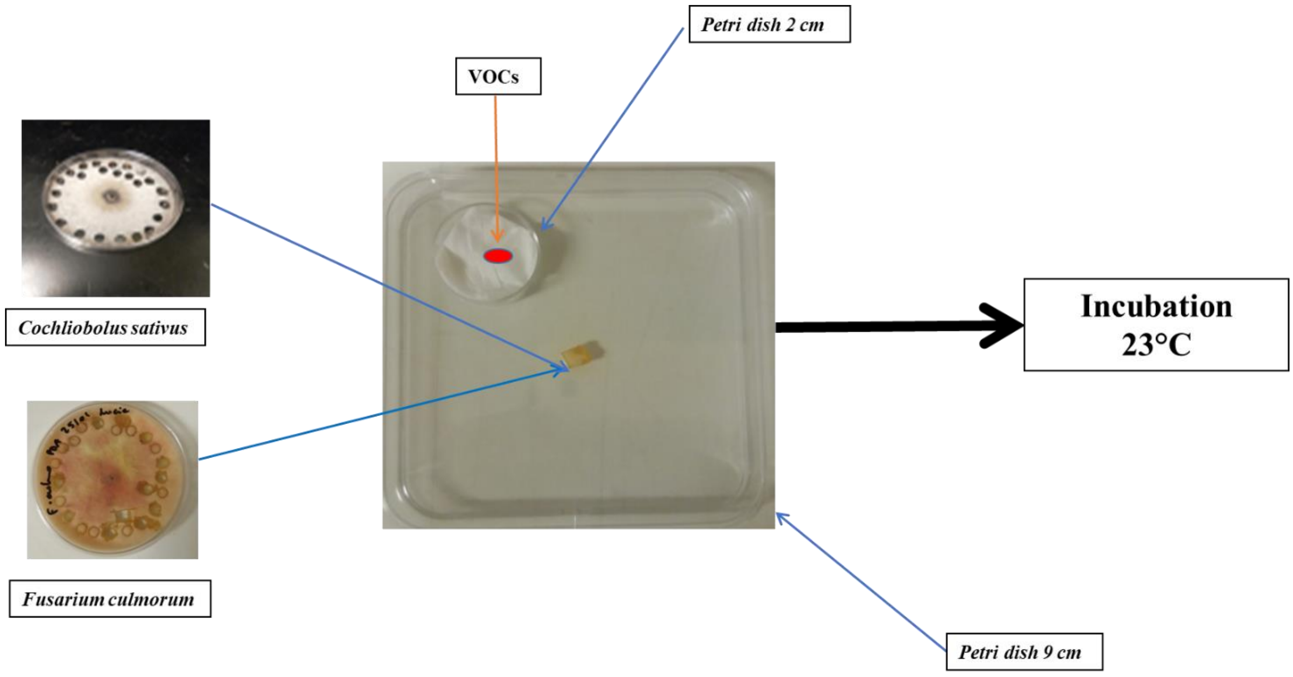

Figure S1. Growth test of C. sativus or F. culmorum in the gas phase with a VOC on water agar or PDA media.

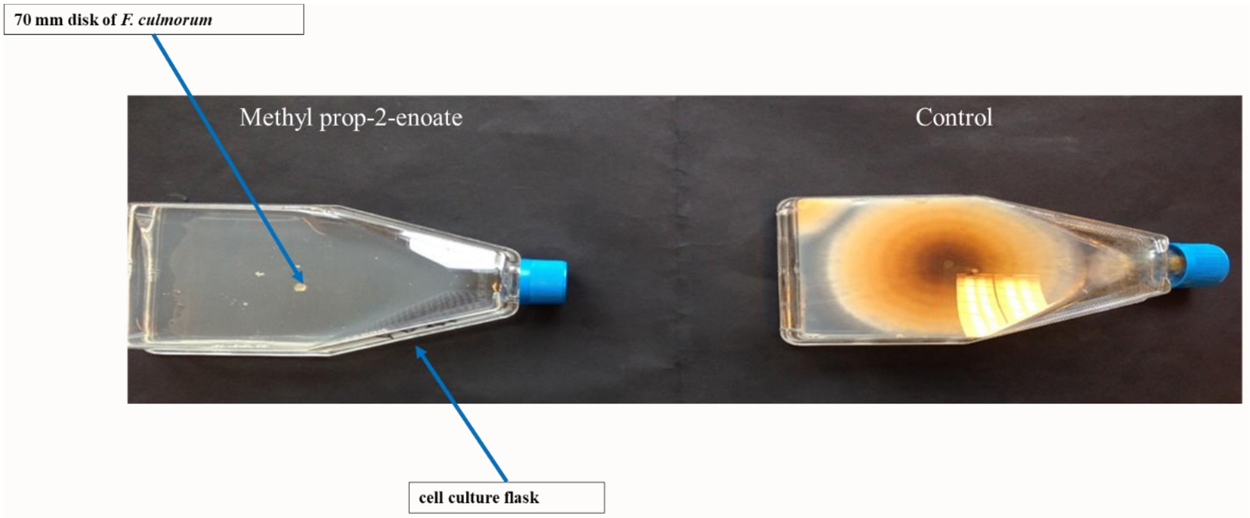

Figure S2. Growth test of F. culmorum in direct contact with methyl prop-2-enoate on PDA media.

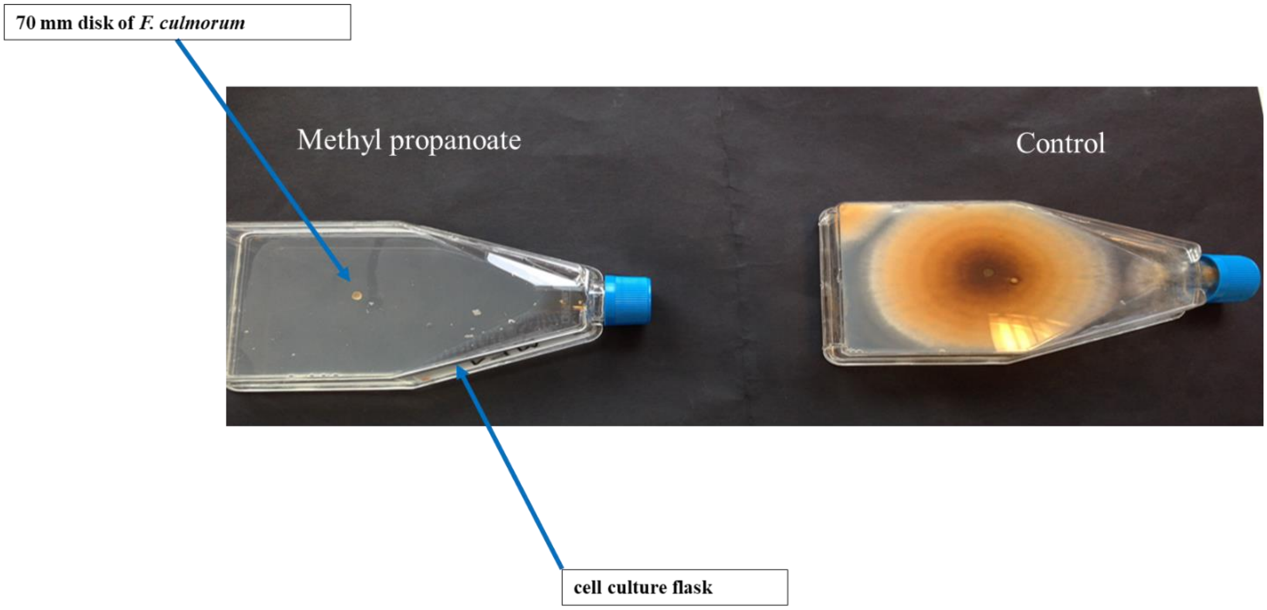

Figure S3. Growth test of F. culmorum in direct contact with methyl propanoate on PDA media.

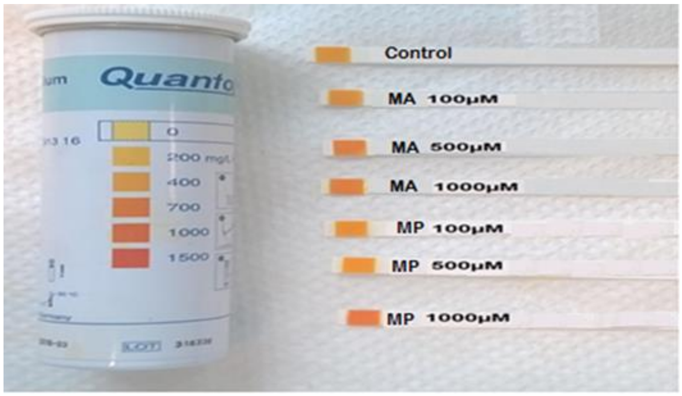

Figure S4. Evaluation of the quantity of K^+ ions released by VOCs treated or non-treated, by conidia of *F. culmorum* after 3 hours

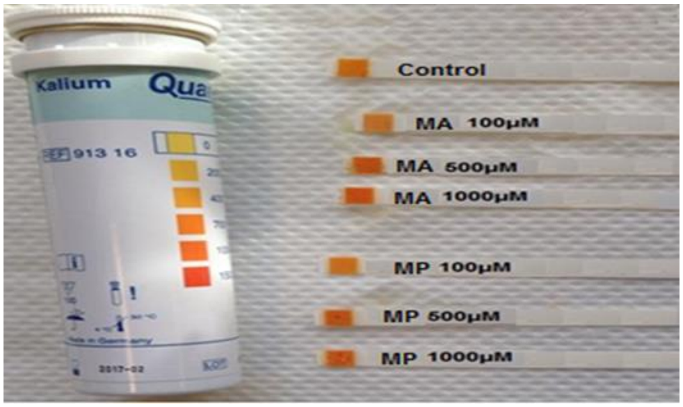

Figure S5. Evaluation of the quantity of K^+ ions released by VOCs treated or non-treated, by conidia *of C. sativus* after 4 hours
